# Supplementary material for: Contribution of paranasal sinus, chest, and abdomen/pelvis computed tomography in patients with febrile neutropenia
Source: PLoS One. 2025 Jan 2;20(1):e0316459. doi: 10.1371/journal.pone.0316459 (PMC11695012; doi:10.1371/journal.pone.0316459)
Supplement: S1 Table — (DOCX) [file pone.0316459.s001.docx]

**Supplementary table 1. Patients’ underlying conditions**

| **HSCT recipients (n=71, 23.2%)** | - Allogenic HSCT : n = 46 (64.8%)  - Autologous HSCT : n = 25 (35.2%) |
| --- | --- |
| **AML and ALL (n=117, 38.2%)** | - Acute myeloid leukemia: n = 99 (84.6%)  - Acute lymphoid leukemia: n = 18 (15.4%) |
| **Lymphoma, CLL and MM (n=52, 17.0%)** | - Lymphoma: n = 46 (88.5%)  - CLL: n = 4 (7.7%)  - MM: n = 2 (3.8%) |
| **MPN and MDS (n=17)** | - MDS: n=16 (94.1%)  - MPN: n = 1 (5.9%) |
| **CAR T-cell therapy recipients (n=24)** | - Lymphoma: n = 22 (91.7%)  - ALL: n = 2 (8.3%) |
| **Solid cancers (n=13)** | - Breast invasive ductal carcinoma: n=4 (30.8%)  - Metastatic melanoma: n=2 (15.4%)  - Metastatic breast cancer: n=2 (15.4%)  - Cancer of unknown primary (CUP): n=2 (15.4%)  - Others: n = 3 (23.1%) |
| **Others (n=12)** | - Aplastic anemia (AA): n=5 (41.7%)  - Inflammatory disease treated with immunosuppressive drugs: n = 3 (25.0%)  - Kidney transplantation: n = 1 (8.3%)  - CTLA4 deficiency: n = 1 (8.3%)  - Hemophagocytic syndrome: n = 1 (8.3%)  - Toxic central cytopenia: n = 1 (8.3%) |

*HSCT: hematopoietic stem cell transplantation; AML: acute myeloid leukemia; ALL: acute lymphoblastic leukemia; MM: multiple myeloma; MPN: myeloproliferative neoplasm, MDS: myelodysplastic syndrome*
