# Supplementary figures and images for: Contribution of paranasal sinus, chest, and abdomen/pelvis computed tomography in patients with febrile neutropenia
Source: PLoS One. 2025 Jan 2;20(1):e0316459. doi: 10.1371/journal.pone.0316459 (PMC11695012; doi:10.1371/journal.pone.0316459)

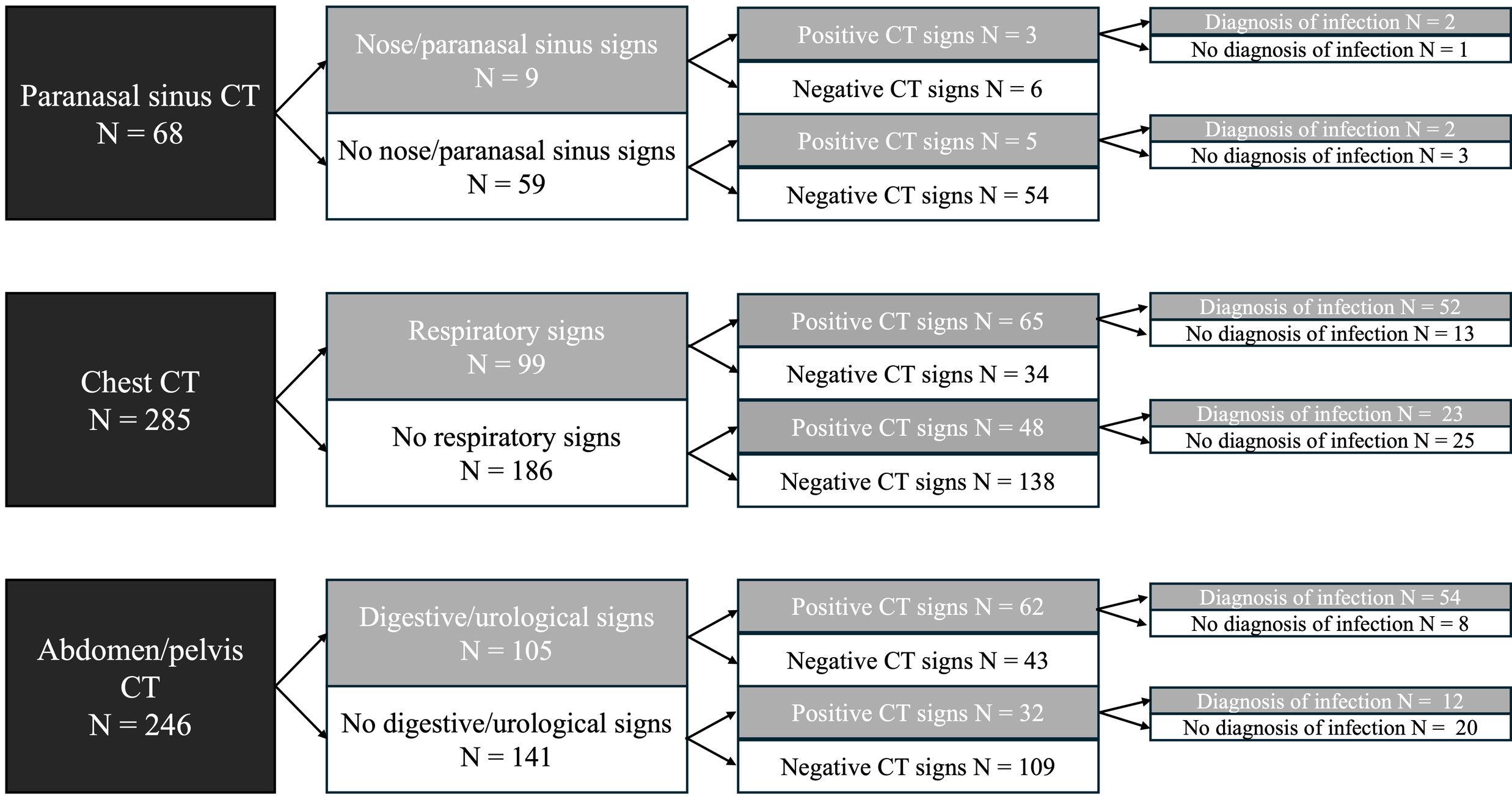

Supplement: S1 Fig — (TIF) [file pone.0316459.s002.tif]

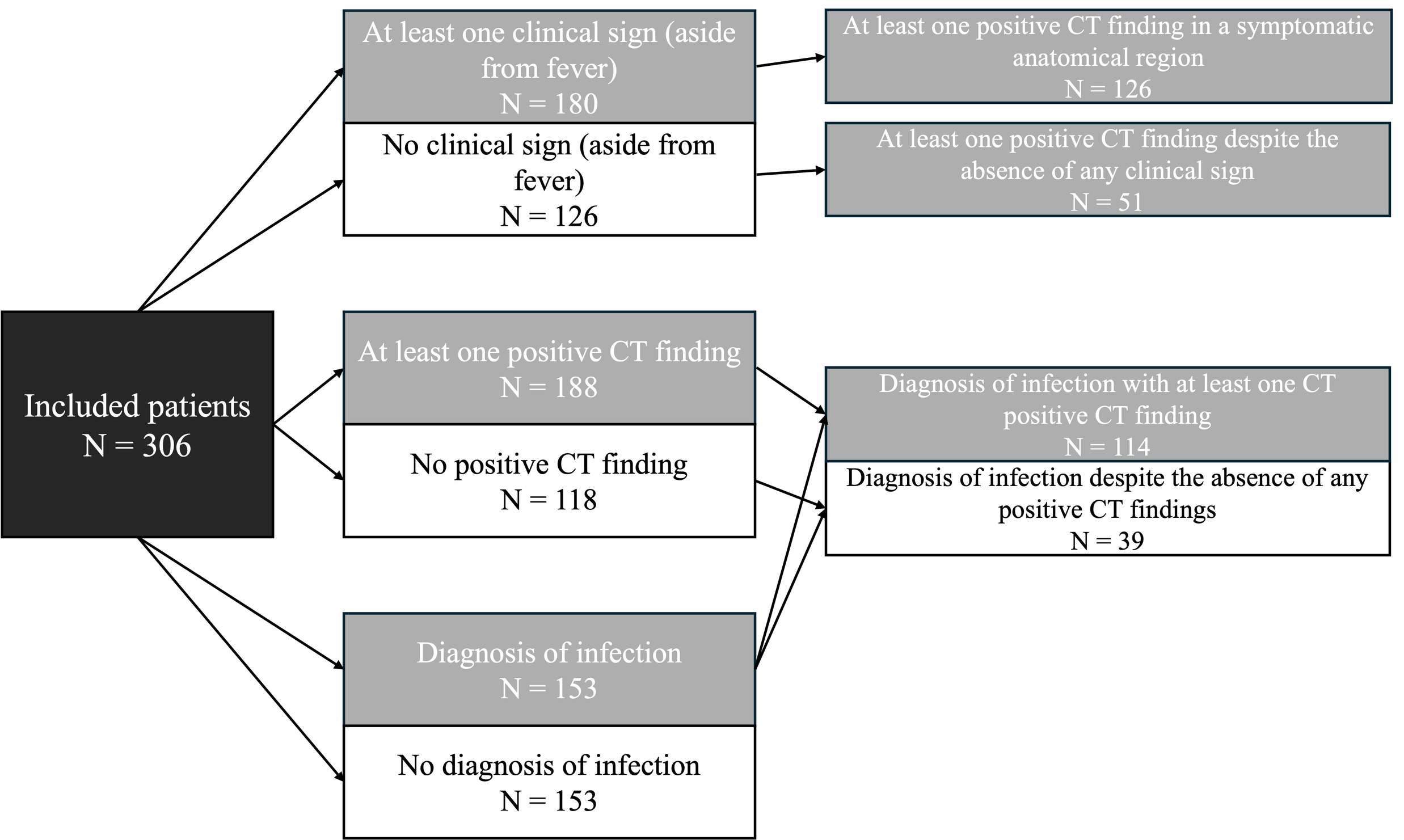

Supplement: S2 Fig — (TIF) [file pone.0316459.s003.tif]
