# Supplementary material for: Contribution of paranasal sinus, chest, and abdomen/pelvis computed tomography in patients with febrile neutropenia
Source: PLoS One. 2025 Jan 2;20(1):e0316459. doi: 10.1371/journal.pone.0316459 (PMC11695012; doi:10.1371/journal.pone.0316459)
Supplement: Attachment — Submitted filename: Response to reviewers.docx [file pone.0316459.s004.docx]

**RESPONSE TO REVIEWERS**

**Journal requirements**

**1.  Please ensure that your manuscript meets PLOS ONE's style requirements, including those for file naming. The PLOS ONE style templates can be found at**

[**https://journals.plos.org/plosone/s/file?id=wjVg/PLOSOne_formatting_sample_main_body.pdf**](https://track.editorialmanager.com/CL0/https:%2F%2Fjournals.plos.org%2Fplosone%2Fs%2Ffile%3Fid=wjVg%2FPLOSOne_formatting_sample_main_body.pdf/1/010f019326564c97-67343c97-d934-4e10-a362-56cfb3e62973-000000/OFE3VtNXbiHb8ysRsetVOAK1PTZAs2fRRf5oktxQfRc=185)  **and**

[**https://journals.plos.org/plosone/s/file?id=ba62/PLOSOne_formatting_sample_title_authors_affiliations.pdf**](https://track.editorialmanager.com/CL0/https:%2F%2Fjournals.plos.org%2Fplosone%2Fs%2Ffile%3Fid=ba62%2FPLOSOne_formatting_sample_title_authors_affiliations.pdf/1/010f019326564c97-67343c97-d934-4e10-a362-56cfb3e62973-000000/7c4Tv13vUf3oKBy-RVREWIKo2sag7N9bEpFCKf-KBQ0=185)

This has been done. Thank you.

**2. Please provide additional details regarding participant consent. In the ethics statement in the Methods and online submission information, please ensure that you have specified (1) whether consent was informed and (2) what type you obtained (for instance, written or verbal, and if verbal, how it was documented and witnessed). If your study included minors, state whether you obtained consent from parents or guardians. If the need for consent was waived by the ethics committee, please include this information.**

**If you are reporting a retrospective study of medical records or archived samples, please ensure that you have discussed whether all data were fully anonymized before you accessed them and/or whether the IRB or ethics committee waived the requirement for informed consent. If patients provided informed written consent to have data from their medical records used in research, please include this information.**

Due to the retrospective nature of the study and because the analysis used only deidentified data (once data collection was done), informed consent was waived by the IRB. We added this information in the Study protocol and patient selection paragraph of the Methods Section.

**3. We note that you have indicated that there are restrictions to data sharing for this study. PLOS only allows data to be available upon request if there are legal or ethical restrictions on sharing data publicly. For more information on unacceptable data access restrictions, please see http://journals.plos.org/plosone/s/data-availability#loc-unacceptable-data-access-restrictions.**

**Before we proceed with your manuscript, please address the following prompts:**

a) **If there are ethical or legal restrictions on sharing a de-identified data set, please explain them in detail (e.g., data contain potentially identifying or sensitive patient information, data are owned by a third-party organization, etc.) and who has imposed them (e.g., a Research Ethics Committee or Institutional Review Board, etc.). Please also provide contact information for a data access committee, ethics committee, or other institutional body to which data requests may be sent.**

According to the current French regulation, authorization to access patient data is given individually to the researcher who has made the request, if this request is judged as acceptable, through the IRB. Therefore, this researcher cannot openly share the data, even anonymized. However, access to data is possible for other researchers on request (aphp-accesdonnees@aphp.fr).

b) **If there are no restrictions, please upload the minimal anonymized data set necessary to replicate your study findings to a stable, public repository and provide us with the relevant URLs, DOIs, or accession numbers. For a list of recommended repositories, please see https://journals.plos.org/plosone/s/recommended-repositories. You also have the option of uploading the data as Supporting Information files, but we would recommend depositing data directly to a data repository if possible. We will update your Data Availability statement on your behalf to reflect the information you provide.**

Please see above.

**Reviewer #1**

**This retrospective study assessed the diagnostic utility of CT imaging across three anatomical regions—paranasal sinuses, chest, and abdomen/pelvis—in 306 patients with febrile neutropenia at a single academic center. The key findings indicate that CT abnormalities were more frequent in symptomatic patients. However, a notable proportion of asymptomatic patients also exhibited positive findings that influenced clinical management, accounting for 25.8% in the chest and 22.7% in the abdomen/pelvis. This evidence supports the routine use of chest CTs in cases of febrile neutropenia and suggests a potential value for abdomen/pelvis CT scans even in the absence of localizing symptoms.

Modification Suggested
1. The study explicitly focuses on immunocompromised patients with febrile neutropenia, excluding those with normal immune function. It is recommended to clearly state in the abstract that the research is centered on the immunocompromised population.**

Thank you for your comment. We believe that, by definition, neutropenia induces a state of immunosuppression. This status can be more or less prolonged, depending on the cause of neutropenia. In other words, a patient with neutropenia does not have a normal immune function. For this reason, we think that mentioning an “immunocompromised population” would be redundant. Most of our patients presented this febrile neutropenia in an onco-hematological context. However, a minority of them had drug-induced neutropenia, in the absence of solid cancer or hematological malignancies (see category “others” in Supplementary table 1).

**2. The methodology section should describe how immunocompromised status was determined for the inclusion of participants.**

The main inclusion criteria included neutropenia with an absolute neutrophile count below 500/mm3, according to the medical files. As a second step, and only for included patients, the primary condition for which patients were being treated when febrile neutropenia occurred was identified from the medical files. The process is detailed in the “study protocol and patient selection” and the “data collection” paragraphs, Materials and methods section.

**These targeted revisions will enhance the manuscript’s scientific rigor and clinical applicability, maintaining the integrity of its core findings and conclusions.**

Thank you for this encouraging feedback.

**Reviewer #2**

**Thank you for sharing this important work on the role of computed tomography (CT) in managing febrile neutropenia (FN) among immunocompromised patients. The study’s focus on imaging as a tool for infection detection in FN patients is timely and clinically valuable, particularly given the complexities of diagnosing infections in neutropenic states. I appreciate the detail and rigor in your methodology, as well as the relevance of your findings for guiding treatment decisions.

Merits
1. High Clinical Relevance: Your study addresses a critical gap in FN management, especially highlighting imaging as a potentially routine assessment tool. For clinicians, the insight into how specific CT findings may influence treatment decisions—particularly chest and abdomen/pelvis imaging—is invaluable.
2. Thorough Methodology: Including multiple anatomical regions and utilizing a structured retrospective analysis strengthens the evidence and reliability of your findings, making it more generalizable to clinical settings with similar patient populations.
3. Significant Practical Implications: By underscoring the influence of positive CT findings on treatment adjustments, you’re providing a basis for possibly expanding imaging practices in FN patients, which could help prevent missed infections or complications.**

We do believe that our work is important in the challenging clinical context of febrile neutropenia, and we thank you for your supportive comments.  **Recommendations for Major Revision
While this study is meaningful, there are areas where further detail and clarification could improve its impact and clarity for readers:**

**1. Provide a Clearer Justification for Paranasal Sinus Imaging: The inclusion of paranasal sinus CT in asymptomatic patients may need more justification. Expanding on any anticipated benefits and clarifying the clinical contexts where it could be useful would add depth and make the rationale clearer for practitioners.**

In our center, paranasal sinus imaging is routinely included in CT examinations of asymptomatic patients, at the request of hematologists, despite the lack of strong scientific evidence on its impact in this population. It is likely due to the fear of fungal infections in high-risk patients, as sinuses are the second most common localization of invasive aspergillosis, with potentially highly severe complication (brain extension especially). Our study is merely an observation of routine practice in our facility. Therefore, we cannot provide a justification for the sinus CT that have been performed. However, as discussed in the Discussion Section, our findings do not provide strong evidence for the benefit of sinus CT in asymptomatic patients. Following your comment, we expanded the discussion on the topic, see the dedicated paragraph in the Discussion section.

**2. Expand on Non-specific CT Findings: It would be helpful to discuss in greater detail the implications of non-specific CT findings, especially in cases without confirmed infection. This could guide clinicians on how to interpret incidental findings and weigh the risks of overtreatment or unnecessary interventions that may arise.**

This a very relevant comment and guiding clinicians in this direction would be very useful. However, our study does not provide data to answer this question, due to its retrospective design, as we now mention in the limitation paragraph of the discussion. We believe that communication between radiologists and referring physicians is key to enable the best treatment option. We added a reference to the importance of this collaboration in the conclusion paragraph of the discussion.

**3. Discuss the Retrospective Design Limitations More Fully: The study’s retrospective nature is mentioned, but providing a more thorough analysis of how this design may impact results—particularly with regard to missing clinical notes or potential biases in treatment decisions—would enhance transparency and contextualize the findings.**

Thank you again. Following this comment and a similar one from Reviewer 3, we developed more this specific point in the Limitation paragraph of the Discussion section.

**4. Address the Systematic Imaging in Asymptomatic Patients: Your findings suggest that systematic imaging may be beneficial even in patients without clinical symptoms. Expanding on existing guidelines or providing a cost-effectiveness perspective on imaging in asymptomatic cases would strengthen the clinical relevance and utility of this recommendation.**

Thank you for this insightful comment. We expanded the 2 corresponding paragraphs in the Discussion section, and we especially added a reference to a very recently published paper (2024) on a similar topic by Chang et al. We could not find, however, any data about cost-effectiveness but we now mention that cost-effectiveness assessment is desirable (conclusion paragraph of the discussion)

**5. Strengthen the Integration of Visual Data with the Text: The tables and figures in the manuscript are informative but could be better integrated within the text. Referring directly to these visuals within relevant sections could enhance readability and make the flow of information smoother for readers.**

We double-checked that all Tables and Figures were called up in the text and included some more explicit references to them.

**Recommendation: Major Revision
In sum, your study has strong clinical implications, and with additional clarification and context in certain areas, it could become a valuable reference for FN management. I appreciate your effort in conducting this research and encourage these revisions to enhance the manuscript’s clarity, accessibility, and relevance for a wide clinical audience.**

**Thank you again for your hard work, and I look forward to seeing the revised version.**

We hope the manuscript has been improved thanks to your comments.

**Reviewer #3: The study investigates the utility of computed tomography (CT) scans across three anatomical regions—paranasal sinus, chest, and abdomen/pelvis—in patients with febrile neutropenia (FN), examining their role in identifying infectious abnormalities and influencing treatment changes. The study addresses an important topic, given the high morbidity and mortality associated with FN and the challenges in identifying infectious sources in this population. The manuscript is well-structured and presents a thorough analysis of clinical, imaging, and therapeutic data in a large cohort. However, there are some areas where the manuscript could benefit from further clarification and refinement.
Minor Comments：**

**1. The authors advocate for routine use of chest and abdomen/pelvis CT in asymptomatic FN patients. While findings suggest that positive CT results are relatively common even in the absence of symptoms, it would be helpful to expand on the clinical relevance and potential cost-effectiveness of this approach. Additionally, providing context with more comparative data from the existing literature could strengthen this argument.**

Thank you for this insightful comment, also made by Reviewer 2. We expanded the 2 corresponding paragraphs in the Discussion section, and we especially added a reference to a very recently published paper (2024) on a similar topic by Chang et al. We could not find, however, any data about cost-effectiveness but we now mention that cost-effectiveness assessment is desirable (conclusion paragraph of the discussion)

**2. Although the authors acknowledge that the study does not evaluate prognosis directly, this is an important point that would benefit from further discussion. For instance, elaborating on how imaging findings might correlate with long-term outcomes or impact clinical decision-making could add depth to the conclusions.**

We totally agree. However, due to the retrospective nature of the study, we do not have data to elaborate on this idea. Previous works have studied the impact of an early diagnosis on the outcome of pulmonary invasive aspergillosis, and we added new reference in the corresponding paragraph of the discussion section.

**3. The findings suggest that paranasal sinus CT had limited impact on treatment changes. The manuscript could benefit from a clearer discussion of whether this justifies excluding sinus CT in the absence of symptoms. Additionally, some comparison to guidelines or prior studies evaluating paranasal sinus imaging in FN patients would provide a stronger basis for these recommendations.**

Thank you for your relevant comment. Following it, and in accordance with Reviewer 2’s similar one, we developed the Discussion paragraph on paranasal sinus CT and added several references. Current guidelines are also mentioned in the same paragraph.

**4. The retrospective nature of the study introduces certain limitations, such as the reliance on available clinical notes for determining symptoms and treatment changes. It would be useful to acknowledge specific confounding factors that may influence the interpretation of CT findings, especially regarding subjective decisions in the clinical diagnosis of infections.**

Thank you again. Following this comment and a similar one from Reviewer 2, we developed more this specific point in the Limitation paragraph of the Discussion section.

**Reviewer #4**

**This study evaluates the effectiveness of paranasal sinus, chest, and abdomen/pelvis CT scans performed during an episode of FN, in patients with or without specific clinical signs to assess potential infectious origin in these sites—their finding support for a systemic CT in these patients with or without symptoms.
This is a reasonable approach, however, as mentioned in the conclusion the potential side effects and high risk of CT which are not essential should be considered.
Several other concerns are noticed in this study:**

**1. In line 73, define other causes of immunosuppression and give all the examples you used in your criteria. The criteria need to be clear and avoid any misinterpretation**

Thank you for your comment. More details about the “others” category are available in the Supplementary Material. However, following your comment, we added the name of the 2 main categories of “others” in the paragraph you mention, and we added the full details of the “others” category in the Supplementary Material, Table 1.

**2. In line 74, define fever (temperature higher than?)**

Thank you. The criterion (oral temperature of >38.3°C or two consecutive readings of >38.0°C) has been added in the mentioned paragraph. **3. In line 78, please clarify why you only considered the first CT.**

We only considered to first CT to keep our data independent and to avoid any clustering within them.  **4. In line 104, please define the physician diagnostic criteria for infectious and non-infectious. What are the lab results and clinical findings in each group?**

It is a very relevant question. However, because of the retrospective nature of the study, we did not have access to the precise criteria on which physicians relied to make their diagnosis. We now mention this point in the Limitation paragraph of the Discussion section.

**5. Line 126-130, when explaining the finding of abdominal CT, make sure to mention any corresponding diagnosis to each of these findings (e.g.: gallbladder thickening and distension correspond to cholecystitis)**

This information is displayed in Table 5 (page 19). Because not all positive findings translated into infectious diagnoses, we decided to only describe CT signs in the lines you mention.  **6. In Table 1, make sure to include the female number % as it might lead to misjudgment of the table data.**

The modification has been done in Table 1 (page 11). **7. Table 4 needs a title heading to be more comprehensive.**

We are not sure to fully understand this comment. However, we changed the title from “etiology of pneumonia” to “microbial etiology of pneumonia”(page 17).
